# 2.5D Deep Learning and Machine Learning for Discriminative DLBCL and IDC with Radiomics on PET/CT

**DOI:** 10.3390/bioengineering12080873

**Published:** 2025-08-12

**Authors:** Fei Liu, Wen Chen, Jianping Zhang, Jianling Zou, Bingxin Gu, Hongxing Yang, Silong Hu, Xiaosheng Liu, Shaoli Song

**Affiliations:** 1 Department of Nuclear Medicine, Fudan University Shanghai Cancer Center, Shanghai 200032, China; liufei0808@126.com (F.L.); sophia_shca@163.com (W.C.); zhangjianping@fudan.edu.cn (J.Z.); gubingxin5117@163.com (B.G.); yhx0610@foxmail.com (H.Y.); husilong007@aliyun.com (S.H.); 2 Department of Oncology, Shanghai Medical College, Fudan University, Shanghai 200032, China; jlzou@fudan.edu.cn; 3 Center for Biomedical Imaging, Fudan University, Shanghai 200032, China; 4 Shanghai Engineering Research Center of Molecular Imaging Probes, Shanghai 200032, China; 5 Key Laboratory of Nuclear Physics and Ion-beam Application (MOE), Fudan University, Shanghai 200433, China; 6 College of Biomedical Engineering, Fudan University, Shanghai 200032, China; 7 Department of Gastrointestinal Medical Oncology, Fudan University Shanghai Cancer Center, Shanghai 200032, China

**Keywords:** 2.5D deep learning, machine learning, PET/CT, diffuse large B-cell lymphoma, invasive ductal carcinoma

## Abstract

We aimed to establish non-invasive diagnostic models comparable to pathology testing and explore reliable digital imaging biomarkers to classify diffuse large B-cell lymphoma (DLBCL) and invasive ductal carcinoma (IDC). Our study enrolled 386 breast nodules from 279 patients with DLBCL and IDC, which were pathologically confirmed and underwent ^18^F-fluorodeoxyglucose (^18^F-FDG) positron emission tomography/computed tomography (PET/CT) examination. Patients from two centers were separated into internal and external cohorts. Notably, we introduced 2.5D deep learning and machine learning to extract features, develop models, and discover biomarkers. Performances were assessed using the area under curve (AUC) and confusion matrix. Additionally, the Shapley additive explanation (SHAP) and local interpretable model-agnostic explanations (LIME) techniques were employed to interpret the model. On the internal cohort, the optimal model PT_TDC_SVM achieved an accuracy of 0.980 (95% confidence interval (CI): 0.957–0.991) and an AUC of 0.992 (95% CI: 0.946–0.998), surpassing the other models. On the external cohort, the accuracy was 0.975 (95% CI: 0.913–0.993) and the AUC was 0.996 (95% CI: 0.972–0.999). The optimal imaging biomarker PET_LBP-2D_gldm_DependenceEntropy demonstrated an average accuracy of 0.923/0.937 on internal/external testing. Our study presented an innovative automated model for DLBCL and IDC, identifying reliable digital imaging biomarkers with significant potential.

## 1. Introduction

Breast lymphoma is a rare extranodal lymphoma that originates from the lymphatic system. Breast cancer is the most common type of malignancy and the second leading cause of death among women worldwide. Breast lymphoma is a rare extranodal lymphoma that originates from the lymphatic system [1,2]. Compared with breast cancer, primary breast lymphoma is relatively rare. Invasive ductal carcinoma (IDC) and diffuse large B-cell lymphoma (DLBCL) are the most common pathological types of breast cancer and breast lymphoma, for which the treatment strategies are completely different. The main treatment methods for IDC include the surgical removal of tumor tissue, radiotherapy, chemotherapy, targeted therapy, and hormonal therapy, however, chemotherapy is the main treatment for DLBCL [3,4,5]. Therefore, it is crucial to accurately distinguish between the two diseases before individualized treatment can be administered.

Previous reviews reported that the symptoms of DLBCL may include breast lumps, breast pain, skin redness, nipple discharge, etc., however, these symptoms may also be indicative of IDC [6,7]. The commonly used diagnostic tools for breast diseases, such as breast X-rays, ultrasound, and magnetic resonance imaging (MRI), all show occupying lesions in the breast area and cannot effectively differentiate between these two types of breast malignancies [8]. Consequently, tissue pathology examination through breast or lymph node biopsy or surgical specimen removal is necessary to diagnose breast IDC and breast DLBCL [9]. However, pathological diagnosis is the gold standard but has risks and drawbacks such as being invasive, prone to infection and bleeding, and insufficient sample size leading to misdiagnosis, etc. [10]. Therefore, it is valuable for the clinic to develop a non-invasive diagnostic tool.

Currently, machine learning (ML) has demonstrated excellent capabilities in processing and analyzing large amounts of heterogeneous medical data and has shown outstanding performance in tasks such as image enhancement, pattern recognition, anomaly detection, predictive modeling, and the formulation of personalized treatment plans. Thus, it has achieved significant results in fields such as medical image diagnosis and clinical decision support for cancer [11,12]. Advancements in artificial intelligence (AI), including deep learning (DL) and ML, in the medical image field have allowed researchers to create accurate prediction models for specific clinical needs [13,14]. It also paves the way for the exploration of digital imaging biomarkers, providing more accurate disease distinction information. This helps in better clinical decision-making and treatment strategy development [15,16]. Employing advanced AI techniques to extract and integrate diverse features could effectively address the challenges posed by the complexity and heterogeneity of data that traditional methods struggle to handle [17,18,19,20]. Ma et al. used the semantic and quantitative features of MRI to establish an ML model to identify benign and non-benign (borderline/malignant) breast nodules and found that five semantic features proved to be significantly correlated with pathological grade [21]. Vanguri et al. developed an ML approach to integrate and synthesize multimodal data routinely obtained during clinical treatment to predict the response to immunotherapy and assess the quantitative expression status and predictive power of biomarkers [22]. Challa et al. recognized imaging and AI-based chromatin biomarkers for the diagnostic and therapeutic evaluation of proton therapy administered to advanced tumors [23]. Furthermore, other studies have shown that AI tools can reveal the relationship between macroscopic images and microscopic genetic information and predict genotypes [24].

To date, in the realm of clinical applications, no diagnostic model supported by AI and grounded in positron emission tomography/computed tomography (PET/CT) has been established for differentiating between breast cancer and breast lymphoma. Drawing on the achievements of our prior end-to-end DL model research [25], we recognize the imperative to push the boundaries of medical image analysis. In the pursuit of more precise disease discrimination, and given the evolving technological landscape, we are now delving into 2.5D DL and ML, integrated with PET/CT radiomics, to distinguish between DLBCL and IDC. This new research not only represents an extension of our previous work, but also holds the potential to improve diagnostic accuracy, bridging the gap between research and clinical applications for the betterment of patient care.

Therefore, the objective of this study was to (i) develop a non-invasive automated diagnostic model for DLBCL and IDC using 2.5D deep learning and machine learning algorithms based on PET/CT radiomics features; (ii) evaluate the diagnostic accuracy, reliability, and generalizability of the developed model across internal and external cohorts for clinical validation; and (iii) identify reliable digital imaging biomarkers associated with DLBCL/IDC discrimination from PET/CT radiomics, providing new imaging evidence for precision diagnosis and treatment decisions.

## 2. Materials and Methods

### 2.1. Patient Selection

**Figure 1:**
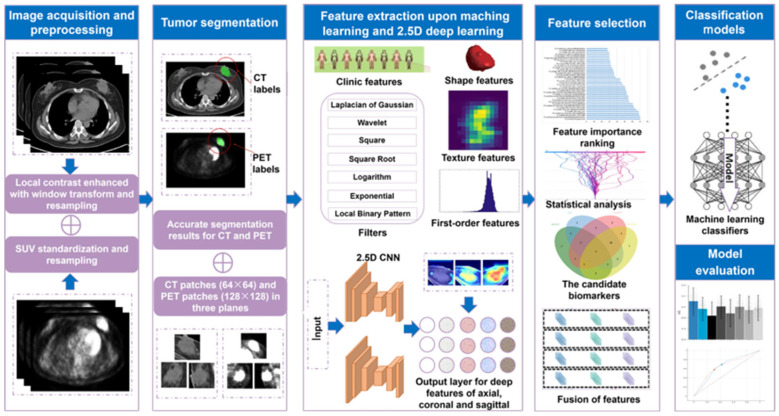
Overview of the study scheme.

**Figure 2:**
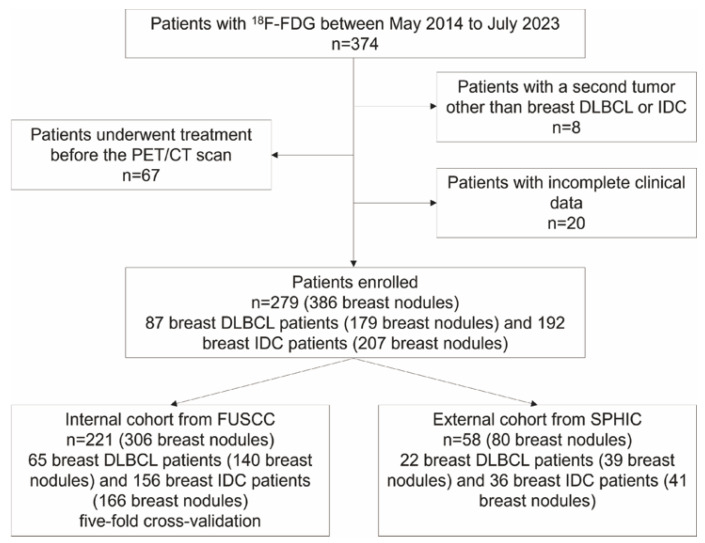
Flowchart of patient inclusion/exclusion.

An overview of our scheme is depicted in Figure 1. All patients meeting the following criteria were enrolled: a definite pathological diagnosis of breast IDC or breast DLBCL, who underwent baseline ^18^F-fluorodeoxyglucose (^18^F-FDG) PET/CT examinations at Fudan University Shanghai Cancer Center (FUSCC) or Shanghai Proton and the Heavy Ion Center (SPHIC), had no prior treatment (radiotherapy, chemotherapy, targeted therapy, or surgery), no secondary primary tumor, and complete clinical data with no loss to follow-up. A total of 279 patients with 386 breast nodules were retrospectively recruited between May 2014 and July 2023. The internal cohort comprised 306 lesions (from 221 patients) from FUSCC, which were divided into training and validation sets using fivefold cross-validation. The external cohort consisted of 80 lesions (from 58 patients) from SPHIC (Figure 2).

### 2.2. Acquisitions and Preprocessing

^18^F-FDG PET/CT scans were conducted using scanners at FUSCC, including Biograph 16HR (Siemnes, Erlangen, Germany), mCT Flow (Siemnes, Erlangen, Germany), uMI 780 PET/CT (United-Imaging, Shanghai, China), and at SPHIC, specifically a Biograph 16 PET/CT (Siemnes, Erlangen, Germany). Patients were required to fast for a minimum of 6 h and maintain blood sugar levels below 10 mmol before receiving an injection of ^18^F-FDG at a dose of 3.7 MBq/kg. Approximately 60 min later, a PET/CT scan from the skull to mid-thigh was performed. Initially, low-dose CT scans were carried out with specific parameters for each scanner (120 kV, 140 mA; 120 kV, 15–100 mA; 120 kV, 150 mA). Subsequently, PET scans were executed and reconstructed using the ordered subsets expectation-maximization algorithm.

PET and CT image resolutions and voxel sizes vary. All CT images were resampled to a 1 × 1 × 1 mm^3^ voxel size and locally contrast enhanced within a range of [−200, 300] Hounsfield units to reduce noise and highlight tumor areas [26]. All PET images were data-converted, with the intensity converted to the standard uptake value (SUV) based on patient weight and resampled to a voxel size of 1 × 1 × 1 mm^3^ [27,28]. This preprocessing enhances the ability to discriminate texture and ensures the comparability and robustness of ^18^F-FDG PET/CT image features across different manufacturers.

### 2.3. Tumor Segmentation

Two attending physicians specializing in nuclear medicine, blinded to the patient’s pathology and with over five years of experience, interpreted the tumor layer by layer. They utilized ITK-SNAP software (version 4.0) to manually segment and label PET images, integrating CT anatomical information. An overlap index was performed to assess the consistency of their segmentation.

### 2.4. Feature Extraction

This study extracted three types of quantitative features. First, clinic features (CFs), which are routine PET metabolic parameters based on nodules including tumor sizes, volume, SUVmin, SUVmean, SUVmax, metabolic tumor volume (MTV), and total lesion glucose (TLG). These were obtained through batch calculation using PyCharm software (v2018.2).

Second, traditional image features (TIFs) are manually extracted from PET and CT scans using a radiomics pipeline. TIFs rely on hand-designed features to describe an image by extracting information such as edges, texture, color, and points of interest. TIFs are categorized into three groups: first-order features that describe the intensity distribution and variability within the lesion; shape features that characterize the geometrical properties of the tumor; and texture features derived from matrices like the gray level co-occurrence matrix, gray level run length matrix, gray level size zone matrix, and gray level dependence matrix [29]. Wavelets and Laplacians of Gaussian features were also used to extract texture features from filtered images. The extraction process for TIFs was implemented using PyRadiomic (v3.1.0), an open-source Python package that adheres to the image biomarker standardization initiative guidelines [30,31].

**Figure 3:**
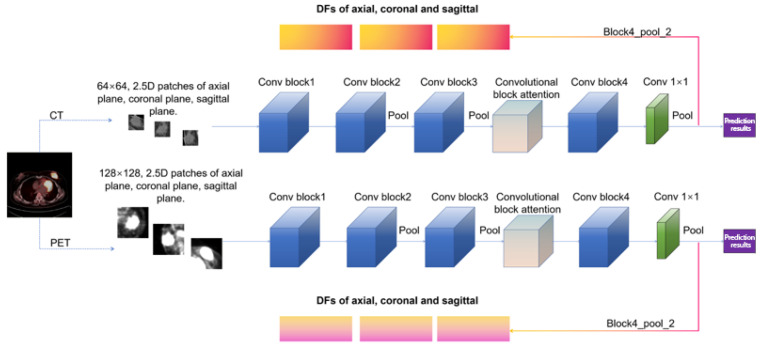
Brief structure and feature extraction of the 2.5D convolutional neural networks.

Third, 2.5D deep features (DFs). For extraction, CT and PET images were cropped based on tumor segmentation results. CT tumor patches were resized to 64 × 64, and PET tumor patches to 128 × 128, for the 2.5D CNN_CT and CNN_PET models. These two networks have the same structure except for the input and are capable of achieving 2.5D feature perception. The schematic diagram of tumor segmentation and the differentiation of DLBCL and IDC by the DL algorithm is displayed in Supplementary Figure S1. There were 54,659 trainable parameters, 23 weighted layers, and 5 pooled layers (Figure 3). We utilized the mish activation function, L2 regularization, dynamic learning rate, and switchable normalization layers [32]. Other hyperparameters and training details are shown in Supplementary Table S1. The last pooling layer “block4_pool_2” (containing 512 channels) in 2.5D CNN_CT and CNN_PET was selected to extract the 2.5D DFs learned by the CNN network from the central slice (containing the largest tumor region) in three different orthogonal planes (axial, sagittal, and coronal planes). For each slice, we used tumor patches as the input to the 2.5D network. The implementation was performed using Nvidia GeForce GTX 1060 and PyCharm software.

### 2.5. Feature Selection

An excessive number of the extracted features above-mentioned may lead to overfitting. It is necessary to reduce their quantity and retain the class-specific features [33]. We utilized four algorithms—max-relevance and min-redundancy, Chi-square, relevant features weighting, and analysis of variance—to reduce and optimize the dimensionality of hybrid features [34,35]. The most critical features were selected to differentiate DLBCL from IDC. This process consists of three steps: (i) determining the number of features; (ii) maintaining key and relevant feature sets; and (iii) calculating the intersection of the feature sets.

### 2.6. Model Development and Evaluation

We developed models using the aforementioned feature sets and their intersections, along with ten embedded ML classifiers in MATLAB (version R2023b). For different types of feature sets (single-modal single-class, single-modal multiclass, multimodal single-class, multimodal multiclass), corresponding ML models were trained. The ML classifiers included decision trees, discriminant analysis, logistic regression, efficiently trained linear classifiers, naive Bayes, support vector machines, k-nearest neighbor, ensemble, neural network, and kernel approximation classifiers. Fivefold cross-validation was applied to the training and validation sets of the internal cohort. The external cohort was used for testing to verify the robustness and stability of the model. Model performance was evaluated by the receiver operating characteristic curve (ROC), accuracy, sensitivity, specificity, and area under the ROC curve (AUC). The positive predictive value (PPV) and negative predictive value (NPV) were calculated using the confusion matrix.

### 2.7. Model Interpretation

We explained the model prediction contributions and decision-making processes using feature importance scores, Shapley additive explanation (SHAP), and local interpretable model-agnostic explanations (LIME). SHAP values reveal global feature contributions based on game theory, while LIME perturbs raw data to observe changes in model predictions. Positive or negative SHAP/LIME values signify positive or negative contributions, respectively.

### 2.8. Statistical Analysis

Next, we ranked the importance of inclusion features, assessed the consistency, and analyzed correlations. Feature importance was evaluated through successive random ranking and scoring. Consistency and correlation analyses were performed using SPSS (version 26.0) to ensure result reliability and understand variable relationships. A consistency value closer to 1 signified greater agreement. Then, we utilized the independent *t*-test, Mann–Whitney U-test for continuous variables, and the Fisher exact test/Chi-square test for categorical variables to compare the inter-group differences. A *p*-value of less than 0.05 was considered statistically significant.

## 3. Results

### 3.1. Study Population

Our study involved 279 patients (277 females and 2 males) diagnosed with breast IDC or breast DLBCL. Demographics in the internal and external cohorts are summarized in Table 1. Among the 279 patients, the average age, height, and weight were 51.39 ± 11.80 y, 1.59 ± 0.05 m, and 59.05 ± 8.70 kg, respectively. Except for weight, there were no significant differences in clinical characteristics between groups. Characteristics between breast IDC and DLBCL are presented in Supplementary Table S2. Of these patients, 192 (68.8%) had breast IDC and 87 (31.2%) had breast DLBCL. For the breast IDC patients, tumor node metastasis stage I, II, III, and IV were 14 (7.3%), 76 (39.6%), 44 (22.9%), and 58 (30.2%), respectively. For the breast DLBCL patients, Ann Arbor stage I, II, III, and IV were 28 (32.2%), 32 (36.8%), 1 (1.2%), and 26 (29.9%), respectively. The difference in clinical stage was statistically significant (*p* < 0.001).

For breast nodules, 306 and 80 nodules were in the internal and external sets, respectively. Nodule characteristics (size and PET parameters) are shown in Supplementary Table S3. The clinical features did not differ between cohorts. In the internal cohort (166 IDC and 140 DLBCL), all characteristics except for SUVmin showed a significant difference between breast IDC and DLBCL (Supplementary Table S4). In the external dataset (41 IDC and 39 DLBCL), only PET parameters (SUVmin, SUVmean and SUVmax) showed a significant difference (Supplementary Table S5).

### 3.2. Feature Analysis

**Figure 4:**
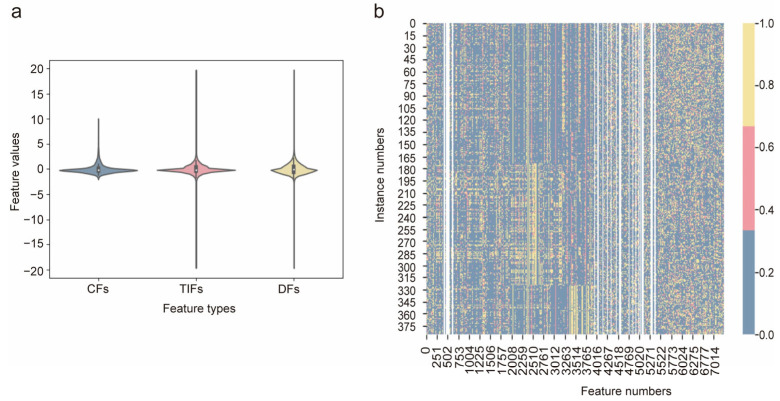
The visualization of selected features including violin plots (**a**) and a heatmap (**b**) for CFs, TIFs, and DFs.

In this study, 7014 quantitative features (8 CFs, 3934 TIFs, and 3072 DFs) were extracted and subjected to Z-score standard deviation normalization. Violin plots and heatmaps were then generated. The violin plot indicated that the CFs and TIFs exhibited dispersed feature distributions, whereas the DFs were concentrated (Figure 4a). The heatmap displays the distribution of all feature values and indicates correlations as well as the role of certain features (Figure 4b). With a sample size of 386, the number of features was set to 38 [36,37,38]. Features were ranked using four selection methods. The top 38 features from the single-modality and single-class were retained, resulting in 16 feature sets (Supplementary Figures S2–S5). The intersection of these feature sets was computed to build an imaging biomarker set. For CFs, features with statistical significance (*p* < 0.05) in both the internal and external cohorts were selected. The feature names and types of candidate non-invasive digital biomarkers are listed in Supplementary Table S6.

### 3.3. Model Performance

**Figure 5:**
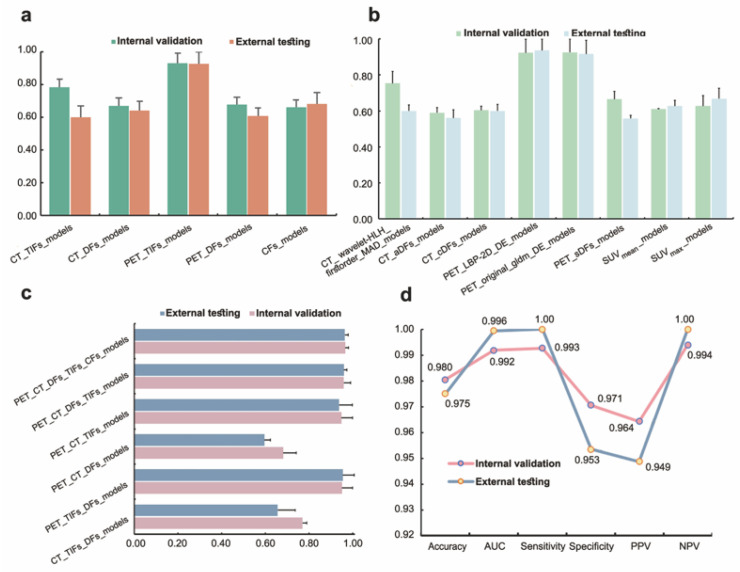
Experimental results for various models: (**a**) average accuracy of models based on single modality, (**b**) average accuracy of models using a single omics of features and candidate non-invasive digital imaging biomarkers, and (**c**) average accuracy of models based on the fusion of multimodel and multiomics features. (**d**) Performance of the PT_TDC_SVM model.

Figure 5a presents a bar graph that illustrates the average accuracy of all models using single-modal and single-class features. The performance metrics for these models were obtained from ten machine learning classifiers applied to sixteen feature sets. Notably, the CT_TIFs feature set achieved an accuracy of 0.784 ± 0.052 (external, 0.579 ± 0.069), CT_DFs reached 0.664 ± 0.048 (external, 0.635 ± 0.056), PET_TIFs had an accuracy of 0.922 ± 0.064 (external, 0.928 ± 0.071), PET_DFs scored 0.668 ± 0.044 (external, 0.660 ± 0.051), and the combined CFs feature set resulted in an accuracy of 0.655 ± 0.047 (external, 0.675 ± 0.068).

Figure 5b illustrates the experimental outcomes of using ten classifiers to model each feature among the set of non-invasive digital biomarker candidates. The internal average accuracies for each feature were as follows: 0.754 ± 0.065 (external, 0.600 ± 0.033), 0.590 ± 0.028 (external, 0.561 ± 0.046), 0.604 ± 0.023 (external, 0.600 ± 0.037), 0.923 ± 0.075 (external, 0.937 ± 0.062), 0.926 ± 0.073 (external, 0.917 ± 0.077), 0.665 ± 0.044 (external, 0.558 ± 0.017), 0.610 ± 0.003 (external, 0.627 ± 0.031), and 0.628 ± 0.057 (external, 0.668 ± 0.058), respectively. Thus, among all of the candidates, the optimal non-invasive digital biomarker was PET_LBP-2D_gldm_DependenceEntropy, and PET_original_gldm_DependenceEntropy could also be considered as an efficient imaging biomarker.

Figure 5c presents the experimental results of combining five feature sets: CFs, CT_TIFs, CT_DFs, PET_TIFs, and PET_DFs. Models utilizing multimodal hybrid features yielded the highest accuracy in distinguishing between DLBCL and IDC, with an average validation accuracy of 0.966 ± 0.015 and an average test accuracy of 0.964 ± 0.016. The subsequent results were as follows: 0.959 ± 0.032 (external, 0.960 ± 0.013), 0.948 ± 0.051 (external, 0.938 ± 0.061), 0.682 ± 0.060 (external, 0.596 ± 0.028), 0.951 ± 0.048 (external, 0.955 ± 0.052), 0.771 ± 0.019 (external, 0.656 ± 0.081).

**Figure 6:**
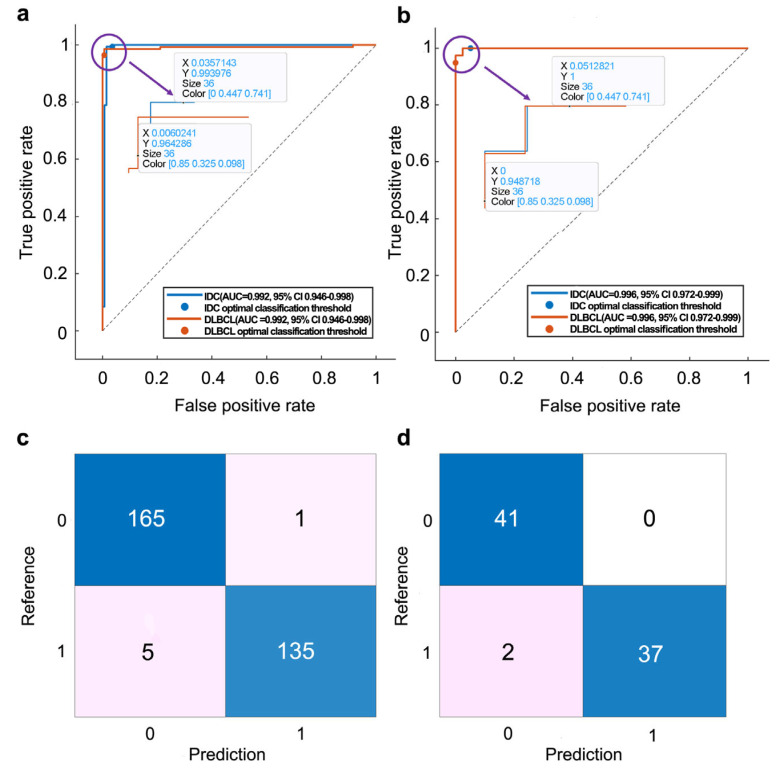
The classification performance of the PT_TDC_SVM model. (**a**) ROC curves for the internal validation and (**b**) external testing sets. (**c**) Confusion matrix for the internal validation and (**d**) external testing sets.

Among all of the multimodal models based on PET/CT with TIFs, DFs, and CFs, PT_TDC_SVM was the optimal choice. The performance line plot is shown in Figure 5d. On the internal validation set, it achieved the highest accuracy of 0.980 (95% confidence interval (CI): 0.957–0.991), the highest AUC value of 0.992 (95% CI: 0.946–0.998), the highest sensitivity of 0.993 (95% CI: 0.954–0.999), highest specificity of 0.971 (95% CI: 0.929–0.989), highest PPV of 0.964 (95% CI: 0.914–0.987), and highest NPV of 0.994 (95% CI: 0.962–0.999). On the independent external cohort, it was 0.975 (95% CI: 0.913–0.993), 0.996 (95% CI: 0.972–0.999), 1 (95% CI: 0.883–1.000), 0.953 (95% CI: 0.829–0.992), 0.949 (95% CI: 0.814–0.991), and 1 (95% CI: 0.893–1.000), respectively. The ROC plot for the PT_TDC_SVM model is shown in Figure 6a,b, and the confusion matrix plot is shown in Figure 6c,d.

### 3.4. Model Interpretation

**Figure 7:**
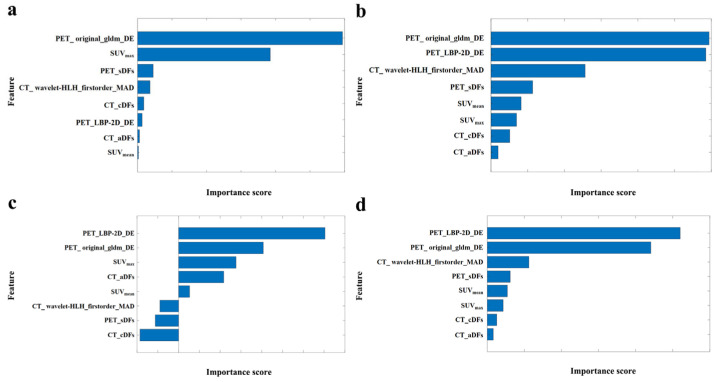
Assessment of feature importance. Feature importance scores are sorted by (**a**) max-relevance and min-redundancy, (**b**) Chi-square, (**c**) relevant features weighting, and (**d**) analysis of variance.

**Figure 8:**
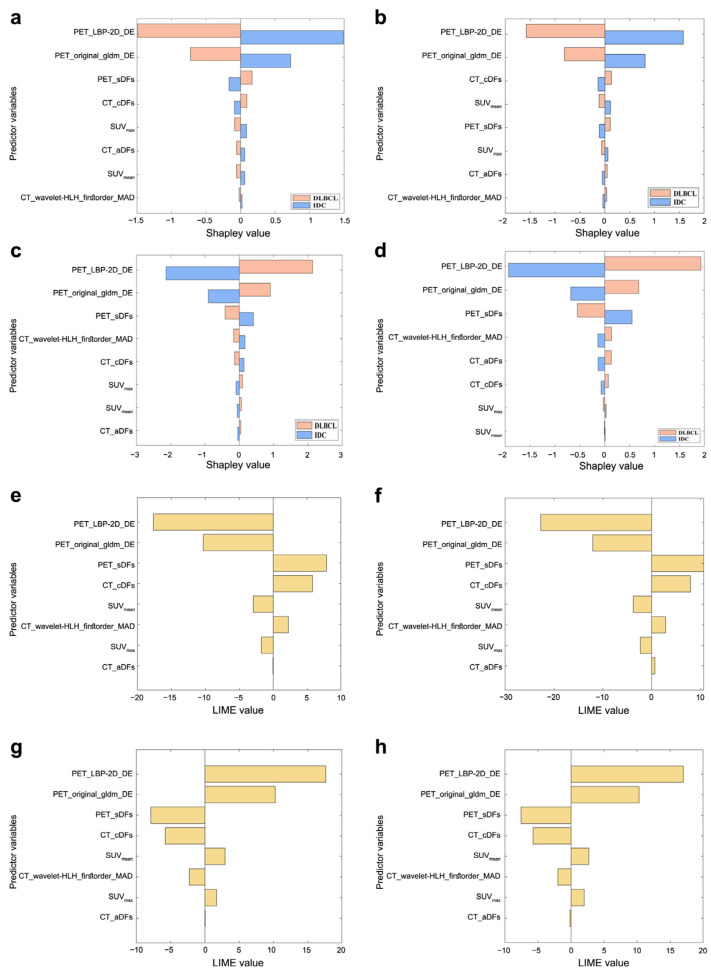
Interpretation of the PT_TDC_SVM model. The global contribution of each feature to the PT_TDC_SVM model output for breast IDC on the (**a**) internal validation and (**b**) external testing, and for breast DLBCL on the (**c**) internal validation and (**d**) external testing. The importance of each feature in the local interpretation for breast IDC on the (**e**) internal validation and (**f**) external testing and for breast DLBCL on the (**g**) internal validation and (**h**) external testing.

Figure 7 shows the importance scores of four algorithms (ranked from top to bottom in descending order), with PET_LBP-2D_gldm_DependenceEntropy and PET_original_gldm_DependenceEntropy being the most critical for distinguishing between the two diseases. Figure 8a,b,e,f shows that PET_LBP-2D_gldm_DependenceEntropy contributed the most to the results when the model predicted DLBCL for each input feature. Similarly, Figure 8c,d,g,h shows its dominant role in IDC predictions.

Feature importance scores further highlight the relative significance of each feature in the PT_TDC_SVM model. While PET_LBP-2D_gldm_DependenceEntropy and PET_original_gldm_DependenceEntropy were the most influential, other features also played certain roles in disease differentiation. Their combination enabled the model to achieve a high prediction accuracy. Using SHAP, we analyzed feature interactions to deepen our understanding of model behavior. The SHAP interaction plots unveiled intricate relationships between features that cannot be captured by feature importance scores alone. Finally, LIME provided a local interpretation of individual predictions, allowing us to observe how each feature contributed to the prediction for a particular input, identify potential outliers or misclassifications, and understand the rationale behind model decisions.

## 4. Discussion

Clinically distinguishing between breast cancer and breast lymphoma is difficult, as is routine imaging diagnosis. Since the treatment methods for these two diseases are completely different, accurately identifying them before treatment is crucial to avoid unnecessary misdiagnosis and surgery [39]. As the gold standard pathological diagnosis for breast disease has limitations like being invasive, having a long processing time, difficulty in obtaining samples, and potential subjectivity and error, there is an urgent need to find a method that can match its diagnostic capabilities for distinguishing between the two diseases [40].

Different AI drivers extract comprehensive multimodal information, which can be combined to enhance system performance. In our study, we developed and evaluated a non-invasive, efficient prediction model for distinguishing between DLBCL and IDC. This was achieved through the integration of multimodal and multiomics features, utilizing 2.5D DL and ML, based on retrospective multicenter and multidevice data analysis. Imaging biomarkers from techniques like MRI, CT, and PET/CT quantify physiological processes and disease states. Quantitative imaging biomarkers offer objective results and aid in personalized treatment [41,42,43]. In our study, we identified, for the first time, two high-sensitivity and specificity imaging biomarkers that differentiated between breast cancer and breast lymphoma.

For breast nodules in the internal cohort, all characteristics except SUVmin showed a significant difference between breast IDC and DLBCL (Supplementary Table S4), consistent with our previous study [25]. 2.5D is a data processing and characterization method that lies between 2D and 3D. While retaining the convenience of 2D data collection, it can avoid the high complexity of complete 3D data and capture the multidimensional structural information of the lesion in 3D space. In this study, for the 2.5D DFs, we extracted sagittal and coronal deep features in addition to axial ones for a more comprehensive understanding [44]. In single modality and single-class feature models, PET_TIFs had the highest average accuracy (Figure 5a) [7,45]. We identified PET_LBP-2D_gldm_DependenceEntropy as the best non-invasive digital imaging biomarker (internal validation accuracy = 0.923, external testing = 0.937) (Figure 5b). PET_original_gldm_DependenceEntropy was similar. Both are related to GLDM dependence entropy, providing quantitative, reproducible, and stable information [46,47,48]. Multimodal and multiomics fusion provides comprehensive tumor information [49]. We established classification models integrating multiple modalities and hybrid features (Figure 5c,d). The PT_TDC_SVM model was the best, with a sensitivity of 1.000 and specificity of 0.954 on the external testing set, which showed significant improvements compared with the previous study (Table 2) [7,25,45]. We then interpreted the output of the PT_TDC_SVM model. PET_LBP-2D_DE was the most important imaging biomarker by various feature importance ranking methods (Figure 7 and Figure 8). This is the first non-invasive digital imaging biomarker with the highest sensitivity and specificity for distinguishing these two diseases.

Regarding future clinical applications, the model developed in this study can be integrated into the existing diagnosis and treatment process in the following ways. First, the model will be packaged as a DICOM plug-in and embedded into the existing hospital PACS system to achieve real-time data interaction with PET/CT equipment. Second, during the practical application of the model, after physicians obtain the patient’s PET/CT images and select the tumor region of interest (ROI), the model will automatically complete tumor feature extraction and classification, generate prompts containing diagnostic probabilities and interpretations of key features, and assist doctors in making decisions. Finally, a lightweight version of the model will be developed to support deployment on low-configuration devices (such as ordinary workstations), thereby lowering the application threshold for primary hospitals.

This study had several limitations. First, as a retrospective study, it was subject to potential selection bias. Therefore, prospective multicenter studies are necessary to further validate the classification models and biomarkers. In the future, we plan to collaborate with tertiary hospitals and primary hospitals to enroll 1000 patients (covering different PET/CT equipment models) to verify the cross-center generalization ability of the model. We will also expand the patient population by increasing the sample size of elderly patients and those with comorbidities, so as to enhance the model’s adaptability to complex cases. Second, despite the dataset expansion, the models for deep feature extraction were trained on small sample sizes and require further optimization. If a series of consecutive 2D slices are taken from the axial, sagittal, and coronal planes, the results might improve from the current state. Third, our current data analysis was solely based on imaging. In the future, we need to integrate multisource data, combining patients’ clinicopathological data (such as immunohistochemical indicators) and follow-up data to construct a multidimensional integrated “imaging-pathology-clinic” diagnostic model. This model will not only enable the differentiation between DLBCL and IDC, but also further assist in the selection of treatment plans.

## 5. Conclusions

In conclusion, we proposed an intelligent classification model based on 2.5D deep learning and machine learning to distinguish breast lymphoma from breast cancer. The model achieved an accuracy of 0.980 and an AUC of 0.992 in internal testing, and an accuracy of 0.975 and an AUC of 0.996 in external testing, which was significantly better than the other comparative models, demonstrating the effectiveness and generalization of the 2.5D strategy. In addition, the optimal imaging biomarkers we screened achieved average accuracies of 0.923 and 0.937 in the internal and external test sets, respectively. These lay a foundation for exploring new diagnostic and therapeutic tools and biomarkers in the future and will contribute to the development of precise tumor diagnosis.

## Figures and Tables

**Table 1:** Patients’ characteristics in the internal and external cohorts.

Characteristics	Total (*n* = 279)	Internal Cohort (*n* = 221)	External Cohort (*n* = 58)	*p* Value
Sex				0.467
Female	277 (99.3)	219 (99.1)	58 (100.0)	
Male	2 (0.7)	2 (0.9)	0 (0.0)	
Age (year)	51.39 ± 11.80	51.58 ± 12.24	51.57 ± 11.47	0.995
Height (m)	1.59 ± 0.05	1.59 ± 0.05	1.58 ± 0.05	0.428
Weight (kg)	59.05 ± 8.70	59.59 ± 8.95	57.00 ± 7.46	0.044
BMI (kg/m^2^)	23.27 ± 3.22	23.44 ± 3.28	22.62 ± 2.95	0.085
Stage				0.281
I	42 (15.1)	29 (13.1)	13 (22.4)	
II	108 (38.7)	90 (40.7)	18 (31.0)	
III	45 (16.1)	35 (15.8)	10 (17.2)	
IV	84 (30.1)	67 (30.3)	17 (29.3)	

Continuous data are demonstrated with the means ± standard deviation or number (percentage). BMI = body mass index.

**Table 2:** Comparison with the recent related study.

Models		AUC	Accuracy %	Sensitivity %	Specificity %	PPV%	NPV%
Ou et al. (CT combined with PT predictive variables) [45]	Internal set	0.85	76.2	86.1	66.7	–	–
	External set	–	–	–	–	–	–
Ou et al. (PETa) [7]	Internal set	0.81	80.8	80.6	84.2	–	–
	External set	–	–	–	–	–	–
Chen et al. (AACNN_E) [25]	Internal set	0.89	83.0	80.9	85.0	84.8	81.2
	External set	0.79	71.6	61.4	84.7	84.0	62.6
PT_TDC_SVM	Internal set	0.99	98.0	99.3	97.1	96.4	99.4
	External set	0.99	97.5	100.0	95.4	94.9	100.0

– not mentioned. AUC = area under the receiver operating characteristic curve; PPV = positive predictive value; NPV = negative predictive value.

## Data Availability

The data and code used to support the findings of this study are available from the corresponding author on request.

## Supplementary Materials

The following supporting information can be downloaded at: https://www.mdpi.com/article/10.3390/bioengineering12080873/s1, Table S1: Hyperparameters and training details of 2.5D CNN; Table S2: Patients’ characteristics between breast IDC and DLBCL; Table S3: Nodule characteristics in the internal cohort and external cohort; Table S4: Nodule characteristics between IDC and DLBCL in the internal cohort; Table S5: Nodule characteristics between breast IDC and DLBCL in the external cohort; Table S6: The candidate non-invasive digital imaging biomarkers; Figure S1: Schematic diagram of tumor segmentation and differentiation of DLBCL and IDC by the DL algorithm; Figure S2: Assessment of single-modality and single-class feature importance. Importance scores sorted by max-relevance and min-redundancy (a), Chi-square (b), relevant features weighting (c), and analysis of variance (d) for the CT_TIFs feature sets; Figure S3: Assessment of single-modality and single-class feature importance. Importance scores sorted by max-relevance and min-redundancy (a), Chi-square (b), relevant features weighting (c), and analysis of variance (d) for the CT_DFs feature sets; Figure S4: Assessment of single-modality and single-class feature importance. Importance scores sorted by max-relevance and min-redundancy (a), Chi-square (b), relevant features weighting (c), and analysis of variance (d) for the PET_TIFs feature sets; Figure S5: Assessment of single-modality and single-class feature importance. Importance scores sorted by max-relevance and min-redundancy (a), Chi-square (b), relevant features weighting (c), and analysis of variance (d) for the PET_DFs feature sets.

## Abbreviations

The following abbreviations are used in this manuscript:

^18^F-FDG	^18^F-fluorodeoxyglucose
AI	Artificial intelligence
AUC	Area under the receiver operating characteristic curve
CFs	Clinic features
CI	Confidence interval
DFs	2.5D deep features
DLBCL	Diffuse large B-cell lymphoma
FUSCC	Fudan University Shanghai Cancer Center
IDC	Invasive ductal carcinoma
LIME	Local interpretable model-agnostic explanations
ML	Machine learning
MRI	Magnetic resonance imaging
MTV	Metabolic tumor volume
NPV	Negative predictive value
PET/CT	Positron emission tomography/computed tomography
PPV	Positive predictive value
ROC	Receiver operating characteristic curve
SHAP	Shapley additive explanation
SPHIC	Shanghai Proton and Heavy Ion Center
SUV	Standard uptake value
TIFs	Traditional image features
TLG	Total lesion glucose
