# Supplementary material for: 2.5D Deep Learning and Machine Learning for Discriminative DLBCL and IDC with Radiomics on PET/CT
Source: Bioengineering (Basel). 2025 Aug 12;12(8):873. doi: 10.3390/bioengineering12080873 (PMC12383681; doi:10.3390/bioengineering12080873)
Supplement: Supplementary file 1 [file bioengineering-12-00873-s001.zip › bioengineering-3764177-supplementary.pdf]

Supplementary Materials

Table S1. Hyperparameters and training details of 2.5D CNN.

Parameters	Specific Settings
Initialization	Uniform distribution of He
Optimizer	Adam (learning rate = 1e-3, β1 = 0.9, β2 = 0.999)
Regularization	L2 weight decay of 1e-4 and Dropout of 0.5
Early Stopping	Stop when validation val_acc does not improve for 30 epochs
Batch Size	64
Data Augmentation	Rescale=1./255; rotation_range=180; width_shift_range=0.2; height_shift_range=0.2; shear_range=0.2; zoom_range=0.2; horizontal_flip=True; vertical_flip=True; samplewise_center=True; samplewise_std_normalization=True.

Table S2. Patients' characteristics between breast IDC and DLBCL.

Characteristics	IDC (n = 192)	DLBCL (n = 87)	p value
Sex			0.564
Female	191 (99.5)	86 (98.9)	
Male	1 (0.5)	1 (1.2)	
Age (year)	51.14 ± 11.76	52.55 ± 12.71	0.365
Height (m)	1.59 ± 0.05	1.59 ± 0.04	0.977
Weight (kg)	58.90 ± 8.67	59.40 ± 8.85	0.654
BMI (kg/m2)	23.20 ± 3.12	23.43 ± 3.46	0.590
Stage			<0.001
I	14 (7.3)	28 (32.2)	
II	76 (39.6)	32 (36.8)	
III	44 (22.9)	1 (1.2)	
IV	58 (30.2)	26 (29.9)	

Continuous data were demonstrated with means ± standard deviation or number (percentage). IDC = invasive ductal carcinoma; DLBCL = diffuse large B-cell lymphoma; BMI = body mass index.

Table S3. Nodules characteristics in the internal cohort and external cohort.

Characteristics	Internal cohort (n = 306)	External cohort (n = 80)	p value
Nodule size			
2D size (cm)	4.43 ± 2.84	4.18 ± 2.52	0.480
3D size (cm)	5.05 ± 3.21	4.90 ± 2.81	0.703
Nodule volume (cm3)	59.18 ± 178.99	44.60 ± 149.35	0.503
PET parameters			
SUVmin	0.60 (0.00, 1.83) *	0.61 (0.01, 1.50) *	0.908
SUVmean	4.95 (1.23, 9.78) *	5.35 (1.33, 24.45) *	0.393
SUVmax	12.79 (2.47, 59.86) *	15.29 (2.45, 59.86) *	0.071
MTV	26.06 (0.20, 815.07) *	15.24 (0.45, 446.06) *	0.294
TLG	224.20 (0.28, 8249.31) *	181.39 (0.80, 7905.91) *	0.707

*Values refer to mean (range), other data is represented as means ± standard deviation. 2D = two-dimensional; 3D = three-dimensional; PET = positron emission tomography; SUV = standard uptake value; MTV = metabolic tumor volume; TLG = total lesion glucose.

Table S4. Nodules characteristics between IDC and DLBCL in the internal cohort.

Characteristics	IDC (n = 166)	DLBCL (n = 140)	p value
Nodule size			
2D size (cm)	3.98 ± 1.98	4.96 ± 3.54	0.004
3D size (cm)	4.49 ± 2.18	5.71 ± 4.02	0.002
Nodule volume (cm3)	23.81 ± 48.08	101.11 ± 253.55	0.001
PET parameters			
SUVmin	0.58 (0.00, 1.79)†	0.63 (0.00, 1.83) †	0.312
SUVmean	3.88 (1.23, 11.84) †	6.20 (1.33, 24.45) †	<0.001
SUVmax	10.08 (2.47, 35.30) †	16.00 (2.49, 59.86) †	<0.001
MTV	10.00 (0.64, 164.63) †	45.11 (0.20, 815.07) †	0.001
TLG	47.22 (1.11, 687.35) †	434.04 (0.28, 8249.31) †	0.001

†Values refer to mean (range), other data is represented as means ± standard deviation. IDC = invasive ductal carcinoma; DLBCL = diffuse large B-cell lymphoma; 2D = two-dimensional; 3D = three-dimensional; PET = positron emission tomography; SUV = standard uptake value; MTV = metabolic tumor volume; TLG = total lesion glucose.

Table S5. Nodules characteristics between breast IDC and DLBCL in the external cohort.

Characteristics	IDC (n = 41)	DLBCL (n = 39)	p value
Nodule size			
2D size (cm)	3.90 ± 1.45	4.48 ± 3.29	0.318
3D size (cm)	4.57 ± 1.63	5.24 ± 3.67	0.302
Nodule volume (cm3)	15.18 ± 17.20	75.52 ± 210.10	0.082
PET parameters			
SUVmin	0.50 (0.03, 1.27) *	0.72 (0.01, 1.50) *	0.012
SUVmean	3.60 (0.99, 7.88) *	7.20 (0.91, 17.72) *	<0.001
SUVmax	10.60 (1.44, 24.80) *	20.23 (1.65, 50.73) *	<0.001
MTV	5.46 (0.45, 40.84) *	25.53 (0.48, 446.06) *	0.112
TLG	22.55 (0.80, 175.67) *	348.37 (0.96, 7905.91) *	0.128

*Values refer to mean (range), other data is represented as means ± standard deviation. IDC = invasive ductal carcinoma; DLBCL = diffuse large B-cell lymphoma; 2D = two-dimensional; 3D = three-dimensional; PET = positron emission tomography; SUV = standard uptake value; MTV = metabolic tumor volume; TLG = total lesion glucose.

Table S6. The candidate non-invasive digital imaging biomarkers.

Features	Subtypes	Image filters	Feature names	Number (s)
CT_TIFs	First Order Statistics	Wavelet	CT_wavelet-HLH-firstorder_MeanAbsoluteDeviation	1
CT_DFs	Axial deep features; Coronal deep features	Convolution kernel	CT_axial_deep_features_450;	2

PET_TIFs	Texture feature	Original; LBP	CT_coronal_deep_features_145 PET_original_gldm_DependenceEntropy; PET_lbp_2D_gldm_DependenceEntropy	2
PET_DFs	Sagittal deep features	Convolution kernel	PET_sagittal_deep_features_497	1
CFs	Conventional biological metabolic parameters	Original	SUVmean; SUVmax	2

CT_TIFs = traditional image features of CT; CT_DFs = deep features of CT; PET_TIFs = traditional image features of PET; LBP = local binary pattern; PET_DFs = deep features of PET; CFs = clinic features.

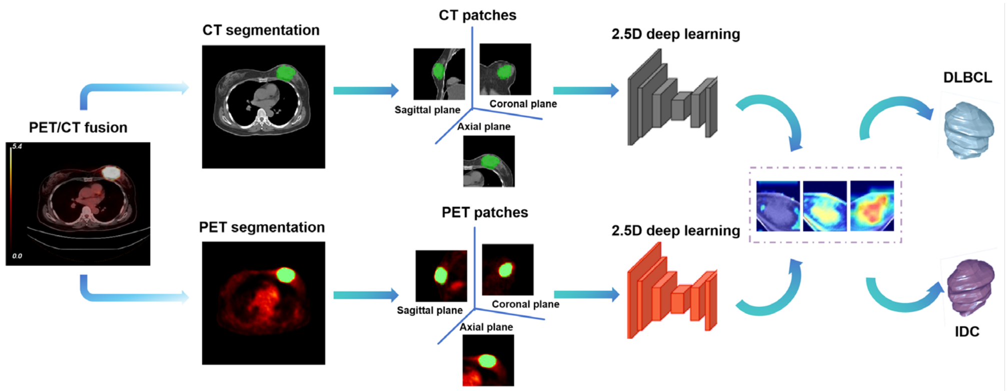

Figure S1. Schematic diagram of tumor segmentation and differentiation of DLBCL and IDC by DL algorithm.

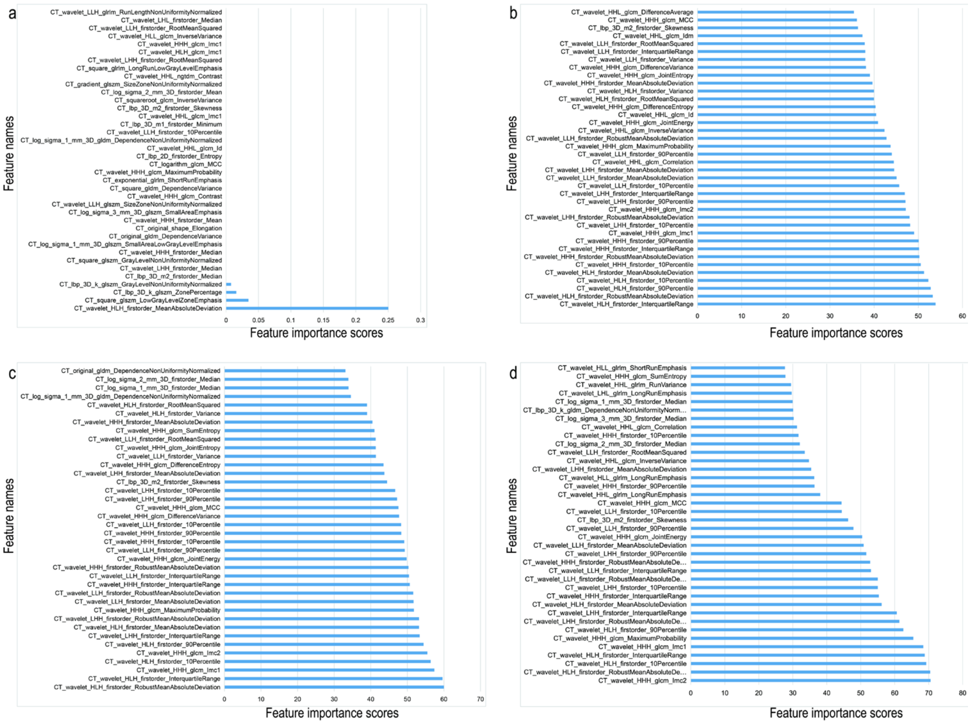

Figure S2. Assessment of single-modality and single-class feature importance. Importance scores sorted by max-relevance and min-redundancy **(a)**, Chi-square **(b)**, relevant features weighting **(c)** and analysis of variance **(d)** for CT_TIFs feature sets.

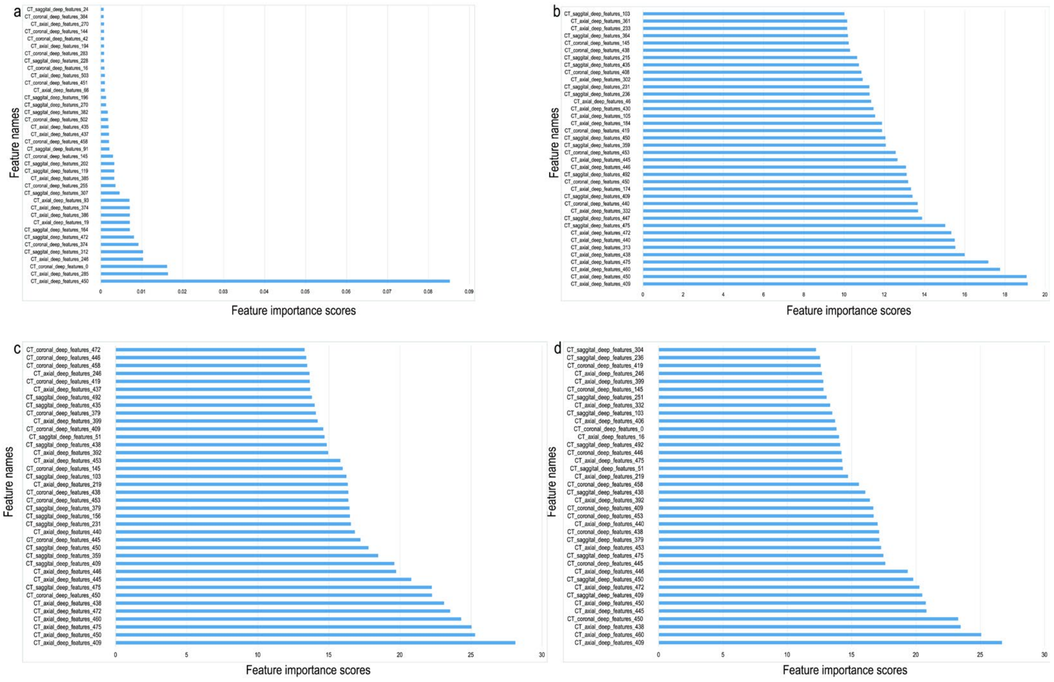

Figure S3. Assessment of single-modality and single-class feature importance. Importance scores sorted by max-relevance and min-redundancy **(a)**, Chi-square **(b)**, relevant features weighting **(c)** and analysis of variance **(d)** for CT_DFs feature sets.

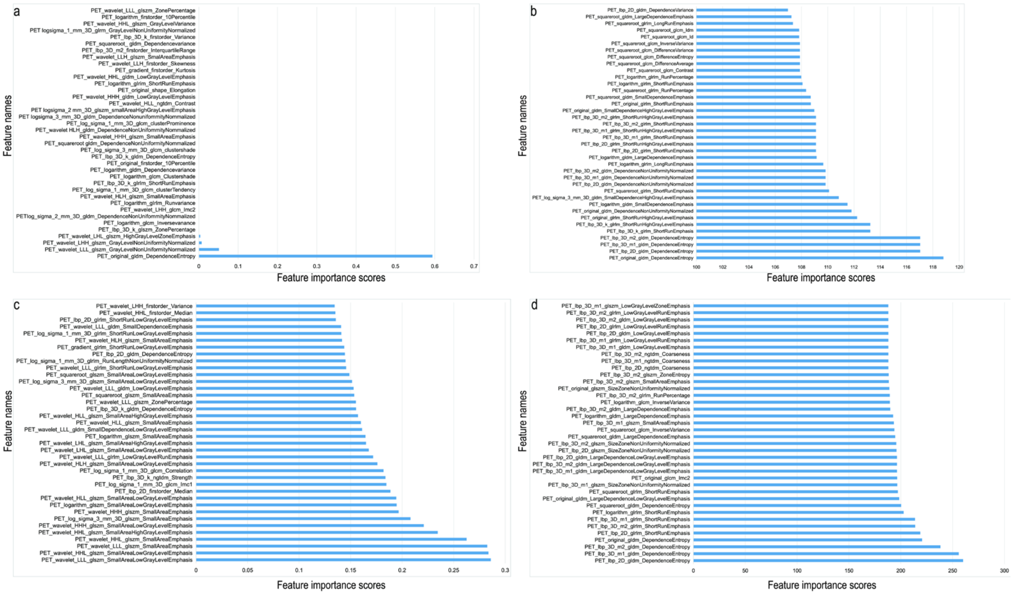

Figure S4. Assessment of single-modality and single-class feature importance. Importance scores sorted by max-relevance and min-redundancy **(a)**, Chi-square **(b)**, relevant features weighting **(c)** and analysis of variance **(d)** for PET_TIFs feature sets.

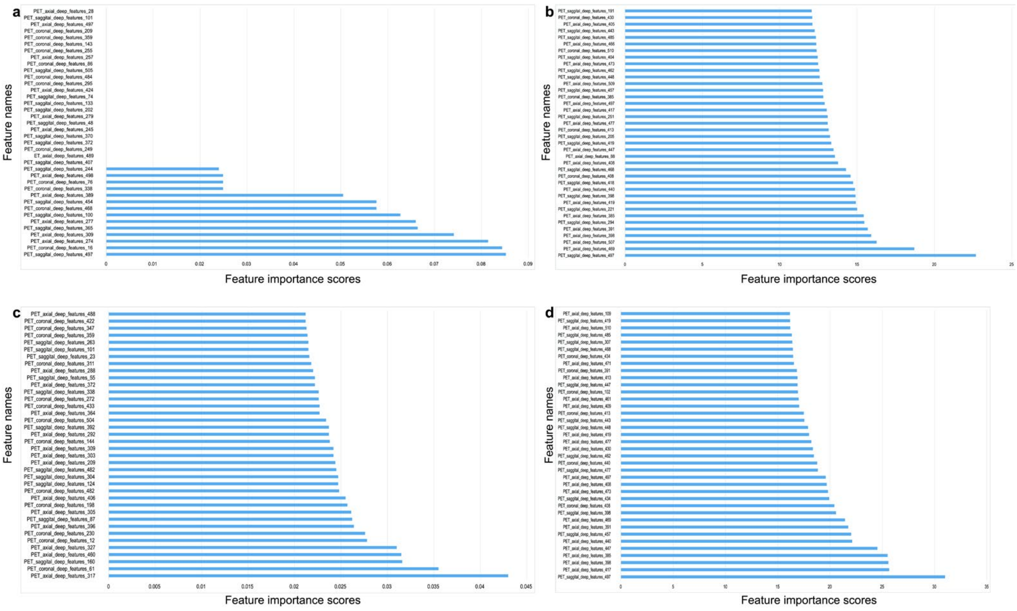

Figure S5. Assessment of single-modality and single-class feature importance. Importance scores sorted by max-relevance and min-redundancy **(a)**, Chi-square **(b)**, relevant features weighting **(c)** and analysis of variance **(d)** for PET_DFs feature sets.
